# Microbial Signatures in Breast Cancer: Exploring New Potentials Across Body Niches

**DOI:** 10.3390/ijms26178654

**Published:** 2025-09-05

**Authors:** Alicia Yoke Wei Wong, Giulia Bicchieraro, Isabella Palumbo, Antonella Ciabattoni, Cynthia Aristei, Roberta Spaccapelo

**Affiliations:** 1 Department of Medicine and Surgery and Center of Functional Genomics (C.U.R.Ge.F), University of Perugia, 06132 Perugia, Italy; aliciayokewei.wong@unipg.it (A.Y.W.W.); giulia.bicchieraro@unipg.it (G.B.); 2 Radiation Oncology Section, Department of Medicine and Surgery, University of Perugia, 06129 Perugia, Italy; cynthia.aristei@unipg.it; 3 Radiation Oncology Section, Perugia General Hospital, 06129 Perugia, Italy; 4 Radiotherapy Unit, San Filippo Neri Hospital, ASL Roma 1, 00135 Rome, Italy; antonella.ciabattoni@aslroma1.it; 5 UniCamillus-Saint Camillus International University of Health Sciences, 00131 Rome, Italy

**Keywords:** breast cancer, microbiota, gut microbiota, breast tissue microbiota, blood microbiota, microbial biomarkers, diagnosis, prognosis

## Abstract

Breast cancer is one of the most frequently diagnosed malignancies and remains the leading cause of cancer-related death among women worldwide. Emerging evidence implicates the microbiota to be a potential contributor to its pathogenesis and progression. This review summarizes emerging evidence of microbial alterations across various body niches in breast cancer patients, including gut, breast tissue, nipple aspirate fluid (NAF), oral cavity, skin, urinary and reproductive tracts, and blood. Reductions in commensal taxa such as *Faecalibacterium*, *Bifidobacterium*, *Lachnospira*, *Akkermansia*, and *Sphingomonas*, along with an increase in pro-inflammatory genera like *Prevotella*, *Fusobacterium*, and *Desulfovibrio*, may promote breast tumor development and progression through multiple pathways including modulation of estrogen metabolism, production of microbial metabolites, and immunoregulation. The presence of cross-niche overlaps and possible translocation of microbiota between niches through the bloodstream suggests the existence of a complex interconnected oral–gut–breast microbiota axis. Progress in the field will depend on integrative multi-omics, translational approaches, and longitudinal studies to give a clearer mechanistic understanding of microbiota–host interactions to develop feasible microbiota-based biomarkers and therapeutic strategies in breast cancer.

## 1. Introduction

Female breast cancer is one of the most frequently diagnosed cancers and is the leading cause of cancer death in women worldwide. The latest available statistics from 2020 report that breast cancer accounted for 2.3 million new cases and almost 670,000 deaths globally [1]. Numerous risk factors have been associated with breast cancer including aging, family history, reproductive factors, estrogen levels, and lifestyle choices [2]. In recent years, evidence has emerged suggesting that the microbiota plays a role in breast cancer. The human microbiota is composed of more cells than human cells in our bodies [3], and the number of its genes vastly surpasses that of human genes [4]. The microbiota functions to potential increase energy extraction from food and alter appetite signaling, thus is involved in metabolic processes [5], provide a physical barrier to prevent the colonization of harmful pathogens through various mechanisms [6], and promote the proper development of the intestinal mucosa and immune system [7]. The role of the microbiota in cancers development, how it affects patient response to therapy, and how it potentially could be harnessed in the clinic is a fast developing field [8]. Although the gut microbiota have been the most comprehensively characterized in the context of breast cancer, emerging evidence is beginning to elucidate the composition and potential functional relevance of microbiota in cancer tissue and additional body niches.

This review summarizes the current literature on microbiota composition changes across various body niches in the context of breast cancer, including emerging niches such as the skin, oral cavity, urine, reproductive tract, and blood. In addition, it explores research on microbial signatures and their potential clinical applications in breast cancer diagnosis and prognosis.

## 2. Changes in the Microbiota of Different Body Niches in the Context of Breast Cancer

Emerging research has highlighted that alterations in the microbiota across various body niches may influence breast cancer development, progression, and treatment responses.

This section explores microbial shifts across different body sites including the gut, breast tissue, skin, oral cavity, female reproductive tract, urine, and blood.

Microbiota changes are commonly assessed through alpha diversity (within sample diversity), beta diversity (between sample differences), and differential abundance analyses to identify significant changes in taxa abundances. In addition, prediction tools and machine-learning models are also used to explore microbial signatures for their diagnostic and prognostic potential [9]. By examining these diverse microbial environments, we aim to better understand their roles in breast cancer and identify new avenues for therapeutic interventions. Table 1 provides an overview of the analytical methods and the geographical origins of patient cohorts in the studies reviewed.

### 2.1. Gut Microbiota

The gut microbiota plays a critical role in maintaining intestinal homeostasis, immune modulation, and metabolic function. Its composition is highly dynamic and influenced by numerous factors such as mode of delivery at birth, infant feeding, age, diet, body mass index (BMI), physical activity, environmental exposures, climate, and geographic location [43]. Furthermore notable differences in gut microbiota composition have been observed between premenopausal and postmenopausal women, a distinction that is particularly relevant given the higher incidence of breast cancer among postmenopausal individuals [10,11,44,45,46]. These factors contribute to the variability in study findings and may explain the limited reproducibility of microbial signatures across investigations.

Several studies have examined gut microbiota diversity in breast cancer, with mixed results. Alpha diversity has been reported as decreased in breast cancer patients compared to healthy or benign controls in some studies [12,13,14,15], while others observed no significant difference [10,11,16,17]. Given that aging and menopausal status are associated with shifts in gut microbiota and breast cancer risk, these variables must be carefully considered when interpreting microbiota data.

Consistent patterns have emerged linking specific bacterial taxa with breast cancer. A reduction in beneficial commensals [47,48,49,50] such as *Faecalibacterium* [11,12,14,16], *Lachnospira* [16], *Bifidobacteria* [11,13], and *Akkermansia* [11,16] has been frequently observed in breast cancer patients gut microbiota. *Bifidobacteria*, widely used in probiotic formulations, has been reported to be 1.9–6.0 times more abundant in premenopausal healthy women compared to those with breast cancer [11,13].

Conversely, potentially pathogenic or pro-inflammatory taxa such as *Prevotella* [10,13] and *Desulfovibrio* [10,17] are enriched in breast cancer patients.

On the other hand, the roles of *Clostridium*, *Fusobacterium*, and *Lactobacillus* in breast cancer remain unclear. *Clostridiales* has been associated with elevated risk of breast cancer [14,15] even if some studies report an increase [12,14] or decrease [13,16] of *Clostridium* in breast cancer patients compared to normal controls. Moreover, specific species vary between studies and findings are inconsistent across menopausal status [17] and tumor grade [18,51]. *Fusobacteria* has been found to be increased in older women with breast cancer compared to age matched healthy controls [10,14], but the contrary was found in premenopausal women [17]. *Lactobacilli*, although generally considered beneficial bacteria, have been found to be both positively and inversely associated with breast cancer depending on the species and hormonal receptor status [10,16]. In addition to the bacteria previously mentioned, several others have been found in increased abundance in breast cancer patients, including *Verrucomicrobia*, *Proteobacteria*, *Actinobacteria* [12]. Specifically, *Citrobacter* [16], *Synergistetes* [17], *Anaerostipes*, and *Bacteroides fragilis* were elevated in premenopausal patients [11], while *Veillonella* was associated with high-grade breast cancer [18].

Few studies reported that gut microbiota composition varies among breast cancer hormonal profiles and subtypes. Estrogen receptor-positive (ER+) patients exhibit increased levels of gut *Megasphaera*, *Roseburia*, *Prevotellaceae* and *Sellimonas*, while patients with ER-negative (ER−) tumors were enriched in *Bacteroides*, *Opitutales*, and other pro-inflammatory taxa [16,52]. Patients with progesterone receptor-negative (PgR−) tumors also show higher levels of *Lactobacillus*, *Acinetobacter*, and *Hydrogenophilaceae*, whereas those with PgR-positive (PgR+) tumors showed enrichment in other taxa such as *Prevotellaceae* (*p* = 0.002) and *Tyzzerella* (*p* = 0.017) [16]. Some gut taxa, such as *Prevotella* and *Clostridiales*, have also been linked to more aggressive molecular subtypes, including ER/PgR/human epithelial growth factor receptor 2 (HER2)-negative (triple-negative (TN)) and Ki-67 high tumors [16]. Distinct gut microbial signature was reported among molecular subtypes, with *Bacteroides* and *Escherichia* elevated in luminal B and HER2+ subtypes, and *Faecalibacterium* lowest in TN breast cancer (TNBC) [19]. However, a study reported no major taxonomic differences between TN and other subtypes, except for reduced alpha diversity in premenopausal TNBC patients [13].

Obesity, a known breast cancer risk factor, is associated with shifts in gut microbiota. Obese women with breast cancer tended to have worse prognosis, have increased treatment complications, and are at higher risk for breast tumor reoccurrence compared to normal weight women [53]. Studies show increased *Firmicutes*, particularly *Clostridiaceae* and *Akkermansia*, in breast cancer patients with higher BMI or total body fat compared to those of normal weight [18,51]. Similar patterns were observed in a mouse model of diet-induced obesity and TNBC [54]. Gut microbiota and adiposity levels regulate hormone bioavailability [55]. Obesity in postmenopausal women has been associated with increased circulating estrogen [56], which can increase their risk of breast cancer [57]. The presence of estrogens and estrogen metabolites in postmenopausal women has been associated with fecal bacteria that are capable of estrogen metabolism, such as the bacteria order *Clostridiales*, as well as the families *Lachnospiraceae* and *Ruminococcaceae* [58]. Early menarche and high adiposity in breast cancer patients have also been linked to lower gut microbial diversity. In a US pilot study, breast cancer patients with early menarche (≤11 years) and high total body fat (≥46%) exhibited reduced diversity, though no specific taxonomic differences were observed likely due to limited sample size (*n* = 37) [18].

Although familial breast cancer accounts for roughly 30% of cases, the gut microbiota relationship with genetic susceptibility remains underexplored. In patients with Phosphatase and TENsin homolog deleted on chromosome 10 (PTEN) Hamartoma Tumor Syndrome (PHTS), an inherited cancer syndrome that predisposes patients to several other cancers, differences in gut microbiota were noted between individuals with and without cancer history, including increased *Rikenellaceae* and *Eubacteriaceae*, and decreased *Bifidobacteriaceae* and *Clostridiaceae* [20].

While accumulating evidence supports the influence of gut microbiota on breast cancer development and progression, study heterogeneity, limited reproducibility, and confounding factors pose significant challenges. Future research should focus on large, well-controlled, and longitudinal studies that incorporate menopausal status, cancer subtype, and host genetic background to better understand microbiome-breast cancer interactions and unravel novel opportunities for diagnostics and therapy.

### 2.2. Breast Tissue Microbiota

Although the presence of microbial components in breast milk has been long established [59], it was only recently confirmed that breast tissue itself harbors a distinct microbiota. The origin of the breast tissue microbiome is still unclear; however, several hypotheses have been proposed. One prevalent theory suggests that microbial translocation may occur via hematogenous routes, whereby bacteria from the oral cavity or gastrointestinal tract disseminate through the bloodstream and colonize mammary tissue [60,61]. Alternatively, commensal microbes from the skin surface may ascend through the nipple ducts, establishing residence within the mammary gland [62]. These proposed mechanisms underscore the potential for multiple sources and dynamic microbial fluxes that collectively shape the breast tissue microbial ecosystem.

Unlike the gut, which is primarily composed of *Firmicutes* and *Bacteroidetes*, breast tissue, both healthy and cancerous, is predominantly colonized by *Proteobacteria* and *Firmicutes* [21,22,23,24,25,26,27,28,63].

Despite some inconsistencies, most studies report significant taxonomic differences between healthy and cancerous breast tissues. While both groups are generally dominated by *Proteobacteria*, subtle shifts in microbial proportions have been observed [22,23]. For instance, one study reported a 6% increase in *Proteobacteria* in malignant versus benign tissues (*p* = 0.0027, Kruskal-Wilcox’s H-test), as well as increased abundances (Linear Discriminant Analysis Effect Size (LefSe), Linear Discriminant Analysis (LDA) score log10 > 2) of taxa such as *Propionicimonas*, *Micrococcaceae*, *Caulobacteraceae*, *Rhodobacteraceae*, and *Nocardioidaceae* [23]. *Sphingomonas yanoikuyae* has also been reported to be enriched in healthy breast tissue compared to tumor tissue [21,24]. The family *Methylobacteriaceae* is also frequently reported, though inconsistently: one study found *Methylobacterium radiotolerans* to be three times more abundant in tumor tissues [21], while another reported a significant decrease in the *Methylobacterium* genus in tumor tissues compared to adjacent normal and healthy tissues [29]. Methodological differences likely contribute to these discrepancies. Studies varied in sequencing protocols (e.g., 16S rRNA V4 region pyrosequencing on formalin-fixed paraffin-embedded (FFPE) tissues [21] versus 16S V3-V4 amplicon sequencing of fresh frozen tissues [29]) and in bioinformatics pipelines for taxonomic classification (e.g., mothur’s Bayesian classifier [21] versus UCLUST [29]). Additional taxa enriched in tumors include *Ralstonia* [24], *Bacillus*, *Staphylococcus*, and unclassified members of *Enterobacteriaceae* [27]. In contrast, beneficial or commensal genera such as *Prevotella*, *Lactococcus*, *Corynebacterium*, *Streptococcus*, and *Micrococcus* were often reduced in cancer patients [27].

When comparing tumor tissue to matched adjacent normal tissue from the same individuals, most studies found no clear separation based on alpha and beta diversity metrics [21,24,27,30]. However, others reported significantly lower total bacterial DNA in tumor samples relative to adjacent tissue [21,24,26,28,30]. A longitudinal study that analyzed breast tissues from healthy controls, individuals who later developed breast cancer (pre-diagnosis), and diagnosed cancer patients found that pre-diagnosis tissues displayed an intermediate microbial profile, suggesting early dysbiosis may precede clinical disease onset [28].

Emerging evidence also indicates that the breast tumor microbiota may be stratified according to hormone receptor (HR) status and molecular subtype. Significant microbial differences were observed between HR-positive and HR-negative tumors. HR-positive samples are enriched in *Paracoccus*, *Actinomyces*, *Hydrogenophaga*, *Halomonas*, *Cutibacterium granulosum*, *Bacillus cereus*, *Staphylococcus aureus*, *Clostridium tetani*, *Acinetobacter baumannii*, and *Spirosoma pollinicola* [22]. Many of these genera are environmental or skin-associated microbes, which may also reflect translocation or colonization dynamics specific to HR-positive tumor microenvironments. In contrast, HR-negative tumors have higher levels of *Acinetobacter*, *Rhodobacter*, *Streptomyces*, *Burkholderiaceae*, and *Priestia megaterium* that potentially can influence the inflammatory milieu [22]. Another study observed that ER-positive tumors had significantly reduced abundances of *Alkanindiges*, *Micrococcus*, *Caulobacter*, *Proteus*, *Brevibacillus*, *Kocuria*, and *Parasediminibacterium* compared to ER-negative tumors [26]. These taxa encompass both commensal and opportunistic organisms, some of which are known to interact with host immune responses or participate in xenobiotic metabolism, suggesting possible roles in modulating tumor progression or response to therapy [55].

Recent studies have also identified specific bacterial genera associated with PgR+ tumors. PgR+ tumors were enriched in *Pelomonas*, *Ralstonia*, *Oblitimonas*, *Lactobacillus*, *Methylophilus*, and *Achromobacter* [26]. Additionally, significant clustering of microbial profiles based on progesterone receptor status was observed using Bray–Curtis dissimilarity (*p* = 0.044), highlighting the distinct microbiome compositions associated with tumor subtypes [64].

HER2– tumors showed increased abundances of *Cloacibacterium*, *PRD01a011B*, *Alloprevotella*, *Stakelama*, *Filibacter*, *Blastomonas*, and *Anaerostipes* and may reflect immune interactions unique to the HER2– tumor microenvironment [26]. In contrast, HER2+ tumors demonstrated a distinct microbial profile, although data remain limited due to smaller sample sizes. For example, one study reported that *Burkholderiales* was more abundant in HER2+ tumors, despite being detected in only four HER2+ samples, suggesting the need for larger cohorts to validate this finding [22]. Additionally, *Pseudomonas* was found to be significantly enriched in HER2+ tumors at the genus level, a genus known for its involvement in immune modulation [19]. Banerjee et al. conducted a comprehensive microbial profiling analysis and identified bacterial signatures that were specific to breast cancer subtypes: HR+/HER2– tumors were associated with *Arcanobacterium*, *Bifidobacterium*, *Cardiobacterium*, *Citrobacter*, and *Escherichia*, many of which are known gut or mucosal commensals; HR–/HER2+ tumors were predominantly associated with *Streptococcus*, a genus that includes both commensal and pathogenic species, some of which have been implicated in inflammatory processes [31].

TNBCs harbor distinct microbial communities compared to other breast cancer subtypes. Multiple investigations have identified an increased abundance of specific bacterial taxa within TNBC tissues, suggesting potential microbiome-driven contributions to their aggressive phenotype. TNBC samples showed increased abundances of several bacterial genera, including *Azomonas*, *Alkanindiges*, *Caulobacter*, *Proteus*, *Brevibacillus*, *Kocuria*, and *Parasediminibacterium* which are typically environmental or opportunistic microbes reflecting possible immunosuppressive microenvironment characteristic of TNBC [26]. Additional taxa reported to be elevated in TNBCs include *Aerococcus*, *Arcobacter*, *Geobacillus*, *Orientia*, and *Rothia*, all of which have been associated with environmental exposure and inflammation. These genera may influence local immune responses or tissue remodeling within the tumor microenvironment [11,13,31]. A study focusing on an Ethiopian breast cancer cohort, further highlighted geographic and population-specific microbial profiles. In this cohort, TNBC tumors showed increased levels of *Burkholderia*, *Thermicanus*, *Paracoccus*, *Mogibacterium*, and *Aeromonas*, suggesting that host genetics, diet, environmental exposures, or regional microbial reservoirs may also shape the tumor microbiome [32]. At the genus level, *Serratia* was consistently found to be more abundant in TNBCs compared to non-TNBCs implicating this organism in potential pathogenic or modulatory roles [19]. These findings collectively underscore that TNBCs are characterized by a unique microbial landscape, distinct from other breast cancer subtypes.

Interestingly, oral bacteria typically associated with periodontal disease have been identified in breast tumor tissues. Tzeng et al. reported elevated levels of *Fusobacterium* and *Porphyromonas*, both linked to poor prognosis, in stage 3 breast tumors [26]. Similarly, Thyagarajan et al. detected *Fusobacterium* in TNBC tissue [37].

Beyond bacteria, some studies have examined the viral component of the breast microbiota. Tumor tissues showed more viral transcripts than healthy tissue and were positive for viruses such as *Epstein–Barr* (EBV) and human *Papillomavirus* (HPV) [22,31,65,66]. It has been proposed that EBV could colonize breast tumors by traveling intracellularly in B lymphocytes, while HPV could have entered the mammary duct through the nipple-areola complex. Both EBV and HPV are proposed to activate oncogenes and cause genetic and epigenetic modulations that can promote breast cancer development [67]. The breast “viriota” also included members of *Retroviridae*, *Herpesvirales* [22,31,33], and *Bracovirus* [22]. Using the PathoChip array, Banerjee et al. also detected various viral, fungal, and parasitic transcripts associated with specific breast cancer subtypes and patient prognoses [31,33].

Collectively, these findings highlight the breast tumor microbiota as a dynamic and subtype-specific feature of breast cancer. Whether these microbial communities actively influence tumorigenesis or simply reflect tumor-associated changes in the microenvironment remains an open question.

### 2.3. Nipple Aspirate Fluid Microbiota

While the majority of studies investigating the breast microbiota in the context of breast cancer have focused on breast biopsy tissues, the microbial composition of nipple aspirate fluid (NAF) is only beginning to be explored. NAF microbiota may arise from both endogenous and exogenous sources, as proposed for breast tissue microbiota more broadly. The dynamic exchange between these microbial reservoirs, along with hormonal, immunological, and anatomical factors unique to the ductal environment, may shape the microbial community structure observed in NAF. Therefore, NAF represents a promising minimally invasive sample type for exploring microbial biomarkers related to breast cancer [68].

In one of the first studies the dominant bacterial phyla in NAF, *Firmicutes*, *Proteobacteria*, and *Bacteroidetes*, mirrored those observed in breast tissue microbiota. Although the overall number of bacterial species did not differ significantly between breast cancer survivors and healthy controls, the microbial community structures clustered distinctly between the two groups. Notably, the genus *Alistipes* was present exclusively in NAF from breast cancer survivors, whereas an unclassified member of the family *Sphingomonadaceae* was more abundant in healthy controls [34]. These findings reinforce previous observations of *S. yanoikuyae* enrichment in healthy breast tissue compared to tumor tissue, suggesting a potential protective or homeostatic role for these bacteria [21,24]. In contrast, another study reported increased alpha diversity in NAF collected from the tumor-bearing breast compared to the contralateral normal breast. They found *Actinobacteria*, *Firmicutes*, and *Proteobacteria* to be the predominant phyla in NAF. Additionally, genera such as *Peptoniphilus* and *Curvibacter* were increased in NAF from healthy and cancerous breasts, respectively [35]. These discrepancies highlight variability between studies and underscore the need for further research to define the NAF microbiota and its relationship to breast cancer pathogenesis. The consistent association of *Sphingomonas* with healthy breast tissue and NAF raises the possibility that this genus may play a protective role.

Overall, these findings emphasize the potential of NAF microbiota as a biomarker source and warrant further investigation into its diagnostic and mechanistic roles in breast cancer.

### 2.4. Skin Microbiota

The breast skin microbiota is the most diverse and least stable compared to other body niches, and it is influenced by a complex interplay of host and environmental factors such as temperatures, humidity, pH, salinity, hormonal status, clothing, sweat and sebum production, all of which influence the composition of skin resident microbes [69]. Despite this complexity, relatively few studies have focused on the microbiota of the skin surrounding the nipple, a region of particular relevance to breast health and disease. Hieken et al. reported that the microbiota of breast skin swabs is distinct from those in underlying breast tissue and breast skin tissue from the same individual. Breast tissue samples exhibited greater species richness and a higher number of low-abundance taxa compared to the skin microbiota, indicating a more complex microbial environment within the tissue itself [36]. In another study, Chan et al. looked at a small number of patients and reported no substantial differences in microbial diversity, composition, or operational taxonomic units (OTUs) between nipple skin and NAF samples when comparing breast cancer patients to healthy individuals [34]. Evidence from these limited investigations are currently insufficient to demonstration if the nipple skin microbiota differs in women with breast cancer, as the study by Chan et al. analyzed breast cancer survivors that already went through breast cancer treatment, that might have influence the microbiota detected [34].

### 2.5. Oral Microbiota

It has been suggested that periodontal disease could be a risk factor for breast cancer, pointing to a possible involvement of oral microbiota dysbiosis in carcinogenesis [70]. However, studies directly investigating the relationship between oral microbiota and breast cancer remain limited. Hieken et al. analyzed oral microbiota from buccal swabs and reported that these microbial communities are distinct from those of breast skin swabs, skin tissue, and breast tissue [36]. However, their study did not assess whether the oral microbiota differed between breast cancer patients and healthy individuals [36]. In a separate study, Wang et al. compared oral microbiota profiles, obtained from saline oral rinses, between breast cancer patients and healthy controls, and found no significant differences in alpha diversity or overall community composition [29].

### 2.6. Female Urinary Tract

Recent studies have begun exploring the urinary microbiota in the context of breast cancer. *Lactobacillus* is the predominant genus in the female urinary tract and plays a key role in maintaining urogenital health. Research has shown that urinary microbiota composition varies with menopausal status, with postmenopausal women exhibiting reduced microbial diversity and shifts in specific taxa. These include a decline in *Lactobacillus* and an increase in genera such as *Prevotella* [71,72], *Garnerella*, *Escherichia-Shigella*, *Atopobium*, *Streptococcus*, and *Dialister* [72]. Wang et al. found that menopausal status, rather than breast cancer diagnosis, was the main driver behind changes in the urinary microbiota. They observed higher abundances of *Corynebacterium*, *Staphylococcus*, *Actinomyces*, and *Propionibacteriaceae* (which are mostly common skin microflora) [69] in urine obtained from breast cancer patients compared to that obtained from non-cancer patients after BMI and menopausal status were taken into account [29].

### 2.7. Female Reproductive Tract Microbiota

The female reproductive tract harbors unique microbial communities that vary in composition and abundance. The lower reproductive tract, particularly the vagina and cervix, hosts the highest bacterial biomass, predominantly composed of *Lactobacillus* species. In contrast, the upper reproductive tract, including the uterus, endometrium, fallopian tubes, and peritoneal fluid, contains approximately four times less bacterial biomass and is characterized by reduced *Lactobacillus* abundance and greater microbial diversity [73]. The microbiota of the female reproductive tract with regard to breast cancer remains largely unexplored. One study conducted in Kazakhstan used a targeted real-time PCR approach to assess 16 vaginal microbial taxa in women with different breast cancer subtypes. The study reported significant differences in the abundance of *Peptostreptococcus* spp., *Lachnobacterium* spp., *Staphylococcus* spp., and *Mycoplasma genitalium* across the subtypes [38]. Breast cancer treatments also appear to influence vaginal microbiota composition. Patients treated with chemotherapy [74] or aromatase inhibitors [75] exhibited a marked reduction in *Lactobacillus* levels. However, these studies are limited by the lack of healthy control comparisons and the use of targeted PCR assays that made it difficult to compare with results obtained from more comprehensive 16S rRNA gene sequencing. Further studies are needed to characterize the vaginal microbiota in healthy women and those with breast cancer, and how the vaginal microbiota changes with breast cancer treatment over time.

### 2.8. Blood Microbiota

Evidence has emerged challenging the notion that blood is a sterile body fluid, showing that there could be microbes present in the circulating blood of healthy individuals. While the composition of microbiota in blood is still debatable, the purported blood microbiota is primarily dominated by *Proteobacteria*, and also contains *Actinobacteria*, *Firmicutes*, and *Bacteroidetes* [76]. Recent studies comparing the blood of more than 9770 healthy individuals supports the theory that blood likely does not have its own microbiota, but instead serves more as a way of sporadic translocation of commensals from other niches within the body [77]. Nevertheless, two studies have comprehensively analyzed the blood microbiota in breast cancer [39,40]. An et al. analyzed a Korean patient cohort through the metagenomic analysis of bacterial extracellular vesicles from patient serum [40], while Peng et al. analyzed the blood microbiota from a Chinese patient cohort and performed integrated microbiome-metabolome analysis [39]. Both studies found that alpha diversity of the blood microbiota was significantly reduced in breast cancer patients compared to healthy controls, and that the beta diversity was significantly different, indicating distinct blood microbiota composition between the two groups [39,40]. An et al. found that breast cancer patients had significantly lower *Pseudomonas*, *Staphylococcus*, *Acinetobacter*, and *Corynebacterium*, and significantly higher *Bifidobacterium*, *Bacteroides*, and *Enterobacter* compared to healthy controls [40]. Peng et al. also found *Bifidobacterium* to be elevated in breast cancer patients, along with *Acinetomyces*, *Clostridium*, *Aquabacterium*, *Campylobacter* and *Methyloversatilis* [39]. Future studies are needed to understand if changes in the blood microbiota of breast cancer patients occur in diverse ethnic and geographical populations. In addition, understanding how detectable microbiota in blood is temporally associated with the presence or absence of disease is crucial before feasible exploitation as a clinical readout.

## 3. Utilizing the Microbiota for Breast Cancer Diagnosis, Prognosis

As research into the human microbiome expands, its clinical utility in oncology is becoming increasingly evident. Microbial communities from the gut, breast tissue, and blood have demonstrated potential for distinguishing breast cancer patients from healthy individuals and for predicting prognosis and treatment response.

Fecal microbiota, given its accessibility and noninvasive nature, has been explored as a diagnostic biomarker. In premenopausal women, *Pediococcus* was more abundant in healthy controls, while *Desulfovibrio* was enriched in breast cancer patients, suggesting these genera could serve as candidate microbial biomarkers for distinguishing between disease states [17]. To improve diagnostic accuracy, machine learning approaches have been applied to microbiome data to classify individuals based on cancer status using pre- and postmenopausal cohorts [10,11,13,17]. The Area Under the Receiver Operating Characteristic Curve (AUC) is often the measure reported to assess the accuracy of diagnosis in these studies where the closer the AUC is to 1.0 or 100%, the closer the test is to perfect discrimination [78]. Studies using fecal microbiota signatures to discriminate breast cancer patients from healthy controls reported AUCs between 72 and 88.7%. The fecal microbiota signatures reported in these studies largely do not overlap apart from *Faecalibacterium prausnitzii* appearing in common in the signatures used (Table 2) [10,11,13]. The results collectively underscore the potential of gut microbiota profiling, particularly when paired with computational modeling, to serve as a noninvasive tool for breast cancer screening and diagnosis across different stages of reproductive aging.

The gut microbiota has also been associated with breast cancer prognosis and treatment outcomes. Yang et al. reported that patients with low Ki-67 expression, often associated with better prognosis, had increased abundance of *Lactobacillus*, *Clostridium*, *Clostridiaceae*, and *Megasphaera*, while high Ki-67 expression correlated with enrichment of *Ruminococcaceae*, *Sporobacter*, and *Tenericutes* [16]. Additionally, tumor-infiltrating lymphocytes (TILs) are considered a marker of favorable prognosis and were associated with greater gut microbial diversity, particularly in HER2+ patients, and correlated with improved response to chemotherapy [41,79].

Breast tissue microbiota has also shown discriminatory potential. Esposito et al. applied a Random Forest model based on microbial features to distinguish tumor from non-tumor tissue with 89% accuracy (Table 2) [30]. Hadzega et al. found that patients with circulating tumor cells (CTC+) exhibited a more diverse microbiota, with increased levels of *Micrococcales*, *Rhodococcus*, *Bacillus*, *Devosia*, and *Moraxella osloensis*, and decreased levels of *Delftia*, *Pasteurella multocida* and certain viruses. Expression of p53 protein, lymphovascular invasion, and nodal status also correlated with specific microbial signatures in breast tissue samples [22]. Tzeng at al. found that lymphovascular invasion was positively associated with *Lactobacillus* but negatively associated with *Oblitimonas* and *Alkanindiges*, while node-positive status was positively correlated with *Acinetobacter* and *Bacteroides* but negatively correlated with *Oblitimonas* and *Achromobacter* [26].

Using microbiota to evaluate breast cancer evolution after drug treatment (chemotherapy, trastuzumab) is also a growing area of research, and there is evidence that microbiota profiling, particularly of the gut, breast tissue, and even oral microbiomes, can be used to monitor treatment response, detect recurrence, or identify residual disease in breast cancer patients. Treatment modalities also influence microbial communities even if little information is available. Chemotherapy has been shown to alter breast milk microbiota composition and reduce diversity [80]. Studies have also found that chemotherapy non-responders exhibit higher abundance of *Bacteroides* and lower levels of *Coprococcus* and *Ruminococcaceae* [81]. In TNBC some bacteria such as *Pandoraea pulmonicola*, *Bacillus sonorensis*, *Brucella melitensis*, and *Legionella pneumophila* differentiated between responders and non-responders [82]. Furthermore, the efficacy of trastuzumab in HER2+ breast cancer patients was diminished following antibiotic use, coinciding with reductions in beneficial gut taxa such as *Lachnospiraceae*, *Prevotellaceae*, and *Coriobacteriaceae* [83].

The potential of using blood microbiota for breast cancer diagnosis was demonstrated by An et al., who analyzed bacterial extracellular vesicles isolated from patient serum. They developed machine-learning models that used bacterial genera as biomarkers to differentiate breast cancer patients from healthy individuals, achieving an excellent AUC of 0.978–0.996 with sensitivities ranging from 0.955 to 0.964 and specificity of 1.000 (Table 2) [40]. While AUC values from microbiome-based models demonstrate promising discriminatory ability, they remain exploratory measures and are not yet validated as clinical diagnostic parameters. Nevertheless, these findings underscore the potential of microbiome signatures to inform prognosis and treatment response once further validated in larger, clinically defined cohorts.

## 4. Emerging Mechanistic Role of the Oral–Gut–Breast Axis in Breast Cancer

**Figure 1:**
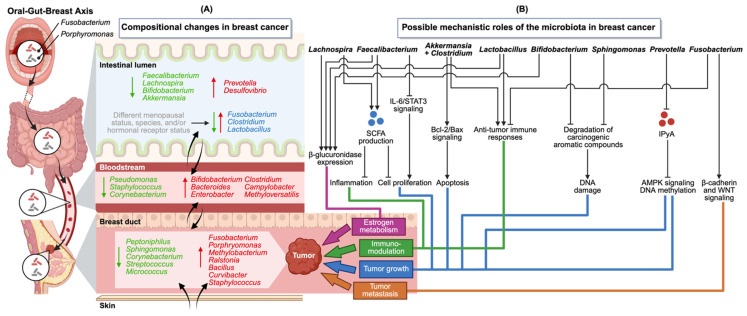
The oral–gut–breast axis microbiota and its possible mechanistic roles in breast cancer. The oral–gut–breast axis proposes a link for how oral-resident bacteria such as *Fusobacterium* and *Porphyromonas* may reach breast cancer tumors. (**A**) Compositional changes (decrease—green, increase—red, increased or decreased—blue) in the microbiota associated with breast cancer have been identified across multiple niches. Cross-niche overlap of taxa suggests possible microbial translocation (as represented by the tapered black arrows). (**B**) Proposed mechanistic roles of key taxa in breast cancer that influence the tumor microenvironment through changes in estrogen metabolism, immunomodulation, tumor growth and metastasis. Commensal genera such as *Lachnospira*, *Faecalibacterium*, *Lactobacillus*, *Bifidobacterium* and *Sphingomonas* may exert protective effects and support anti-tumor immunity. In contrast, pathogens or opportunistic microorganisms such as *Prevotella* and *Fusobacterium* may promote tumor growth and metastasis. Abbreviations used: AMPK, AMP-activated protein kinase; Bcl-2, B-cell lymphoma 2; IL-6, interleukin-6; IPyA, indole-3-pyruvic acid; SCFA, short-chain fatty acid; STAT3, Signal Transducer and Activator of Transcription 3.

Observations of cross-niche overlaps and possible translocation of microbiota between niches through the bloodstream suggests the existence of a complex interconnected oral–gut–breast microbiota axis [19] that may influence breast cancer development and progression locally or distally (Figure 1).

Commensal genera such as *Faecalibacterium* [11,12,14,16], *Lachnospira* [16], and *Akkermansia* were generally decreased in the gut in breast cancer patients (Figure 1A). *Faecalibacterium* and *Lachnospira* are known producers of anti-inflammatory metabolites including short-chain fatty acids (SCFAs), butyrate [50] and propionate [17] which have demonstrated antiproliferative effects on breast cancer cells. *F. prausnitzii* supernatants have been shown to inhibit interleukin-6/Signal Transducer and Activator of Transcription 3 (IL-6/STAT3) signaling, which in turn led to an inhibition of breast cancer cell growth, pointing to a possible mechanism how gut resident *F. prausnitzii* is able to influence distal tumor cells [12]. *Akkermansia muciniphila* together with *Clostridium butyricum* has been demonstrated to inhibit breast cancer progression in pre-clinical models through the activation of anti-tumor immunity through increasing Tumor Necrosis Factor-4 (TNF-4) production and CD8^+^ lymphocyte infiltration, while promoting apoptosis through the B cell lymphoma 2 (BCL2)/BAX pathway [84]. While the role of *Lactobacillus* in breast cancer is not currently clear, mechanistically *Lactobacillus* may influence breast cancer progression via modulation of immune responses and cytokine production. In preclinical models, oral administration of *Lactobacillus reuteri* significantly suppressed mammary tumor growth (*p* < 0.01), attributed to increased CD8+ T cell infiltration and anti-inflammatory cytokine expression [85]. Several microbes are known to degrade environmental polycyclic aromatic hydrocarbons (PAHs) which can cause malignancy by binding to DNA, RNA and protein, causing damage that can lead to mutations [86]. *Bifidobacterium* was also decreased in gut microbiota in breast cancer [11,13]. *Bifidobacterium* spp. such as *Bifidobacterium longum* have shown anti-cancer effects through unknown mechanisms [87], decrease mutagenicity of benzo[a]pyrene, and bind to carcinogenic aromatic compounds [86]. *Sphingomonas*, on the other hand, was consistently associated with healthy breast tissue and NAF raises the possibility that this genus may also play a protective role. Species closely related to *Sphingomonas*, such as *S. yanoikuyae*, are known to degrade aromatic compounds, providing a plausible mechanism for their protective effect (Figure 1B) [88,89].

On the other hand, pathogenic or opportunistic bacteria such as *Prevotella*, *Desulfovibrio*, and *Fusobacterium* are generally upregulated in the gut in breast cancer patients, or in breast tumors (Figure 1A). *Prevotella copri* has been found to promote tumor growth, in mice, through the reduction in the tumor-suppressing metabolite indole-3-pyruvic acid (IPyA), that inactivates AMP-activated protein kinase (AMPK) signaling and DNA methylation pathways. *Desulfovibrio* abundance was suppressed in mice treated with paclitaxel, suggesting a role in disease progression [90]. *Fusobacterium nucleatum*, a known oncogenic bacterium in colorectal cancer, may also contribute to breast cancer progression by modulating immune responses and promoting metastasis in pre-clinical models [60]. *F. nucleatum* is able to promote cancer through various mechanisms such as its ability to travel to distal tumors via the bloodstream, attaching to tumor cells overexpressing the glycan D-galactose-β(1-3)-N-acetyl-D-galactosamine (Gal-GalNAc) via the lectin Fap2 [60], as well as modulation of inflammatory signaling and the promotion of oncogenic WNT signaling through Fusobacterium adhesin A (FadA) that can promote tumor proliferation and metastasis (reviewed in Little et al.) (Figure 1B) [91]. These findings raise the possibility of microbial translocation or systemic inflammatory pathways connecting oral and breast tissue health.

β-glucuronidase is an enzyme that increases free-form estrogen by participating in deconjugation of bound estrogen [92]. Elevated circulating free-form estrogen in postmenopausal women have been associated with increased breast cancer risk [57,93]. Given that numerous risk factors associated with breast cancer are also linked to estrogen metabolism, bacteria that express β-glucuronidase might possibly play a role in breast cancer development and progression as well. The β-glucuronidase gene has been shown to be expressed in members of *Lachnospiraceae* and *Ruminococceae* [94], as well as other bacteria such as *Bifidobacterium*, *Alistipes*, *Coprococcus*, *Faecalibacterium*, and *Lactobacillus* (reviewed in Hu et al.) (Figure 1B) [95]. However, the exact contributions to these β-glucuronidase-expressing bacteria to regulation of estrogen levels and the relation to breast cancer risk remains to be clarified and thus warrants further investigation.

Additionally, murine models show that microbiota can influence mutated p53 activity through metabolites such as gallic acid, highlighting the potential interplay between host genetics and microbial metabolism [96].

The functional significance of these microbes, whether they actively contribute to tumor progression, modulate local immune responses, or simply reflect underlying tumor biology, remains to be fully elucidated.

## 5. Challenges and Prospects for Clinical Translation of Microbiota Findings

Substantial progress has been made in understanding the role of the microbiota in breast cancer. Numerous studies have suggested that microbial signatures may contribute to diagnosis, prognosis, and therapy response prediction. However, translating these findings into clinical practice remains challenging due to a combination of biological, technical, and translational limitations that confound findings and contribute to inconsistencies between studies (summarized in Filardo et al., and McGuinness et al.) [97,98].

Biological and population-related heterogeneity represents one of the major challenges in microbiota research related to breast cancer. Microbiota composition is influenced by multiple host and environmental factors, including geography, diet, ethnicity, menopausal status, BMI, in both in healthy individuals and those with tumor subtype [99]. For example, Hou et al. reported higher prevalence of premenopausal breast cancer in Asian cohorts compared to Western cohorts [11], which may further complicate cross-study comparisons due to age- and hormone-related microbial variations. Other studies have shown distinct tumor-associated microbiota in patients of different racial backgrounds, even within the same geographic region [37]. Most data are derived from populations in Western, Educated, Industrialized, Rich, and Democratic (WEIRD) countries. Furthermore, most microbiota studies have been conducted in ethnically homogeneous cohorts with underrepresentation of low- and middle-income countries, making it challenging to establish globally relevant microbiota-based biomarkers.

Another major limitation in microbiota studies is small sample size due to recruitment difficulty, cost, and the complexity of multi-omics workflows. This issue is even more evident when cohorts are subdivided by tumor subtype, receptor status, age, parity, or BMI. Consequently, most studies choose to focus on a few variables. In the case of BMI, it has been demonstrated that differences in microbiota can occur if BMI categories were too broad [100]. These limitations reduce statistical power and increase the risk of overfitting, particularly in machine learning models. Therefore, meta-analysis could also serve as a valuable approach to mitigate the problem of small sample sizes by integrating data from multiple studies, allowing for more robust and comprehensive analyses. Meta-analyses, such as those of Thu et al. [101] and Luan et al. [102], could potentially overcome this problem but, at the same time, reflect the difficulties in cross-study comparisons due to variations in methodologies, sample processing, variables analyzed, and data interpretation.

Additional and critical limitations are linked to variability in protocols, from sample collection to analysis, limiting reproducibility and cross-study comparison. For example, breast tissue and NAF are a low-biomass sample types, increasing the risk of contamination from reagents or environmental sources, hence care should be taken to address potential contaminants in microbiota data as Chan et al. did [34]. The use of FFPE tissue, common in retrospective studies, can degrade DNA and introduce sequencing artifacts. Fresh-frozen tissue provides better-quality DNA but, sometime, it is more logistically complicated to achieve [103]. Moreover, definitions of healthy controls vary broadly, including adjacent normal tissue [21,24,28,30,36,37], reduction mammoplasty tissue [21,22,29], or donor samples [25,26,31,33], each with potentially distinct microbial baselines. Furthermore, discrepancies in microbial profiles across studies are frequently related to sequencing protocols, platforms used and bioinformatic pipelines. Most studies rely on 16S rRNA gene sequencing but differ in their choice of hypervariable region [10,13,24,25] (e.g., V3-V4 vs. V4, mostly the Illumina platform) (Table 1), primers, and DNA extraction methods. These technical differences can profoundly affect taxonomic resolution and bias abundance estimates. Indeed, Costantini et al. [24] and German et al. [25] have shown that different regions of the 16S rRNA gene can lead to differences in taxa detection. Zeber-Lubecka et al. [13] and Zhu et al. [10] both used shotgun metagenomic sequencing to analyze microbiota differences between pre- and postmenopausal breast cancer patients, which should have higher resolution on the bacteria species level. Nevertheless, they identified different taxa due to geographically cohort differences, updated bioinformatic tools, and variations in reference databases. Variability in bioinformatic pipelines (e.g., mothur, Quantitative Insights Into Microbial Ecology (QIIME), Kraken, Metagenomic Phylogenetic Analysis (MetaPhlAn3), UCLUST, Divisive Amplicon Denoising Algorithm 2 (DADA2), Illinois Mayo Taxon Organization from RNA Dataset Operations (IM-TORNADO)) and the choice of reference databases (e.g., Greengenes, SILVA, Ribosomal Database Project (RDP)) across laboratories can introduce inconsistencies in taxonomic classification and downstream microbial community analyses (Table 1). While standardization is desirable, it is not always feasible due to evolving technologies, platform availability, and funding disparities [97].

Most studies to date have focused exclusively on bacterial communities. Technologies capable of detecting more than bacteria in the microbiome such as viruses, fungi and archea is only just starting to be used. For example, Banerjee et al. used the PathoChip platform to detect microbial and viral transcripts in breast tumors, while Hadzega et al. and Huang et al. used RNA-seq and internal transcribed spacer (ITS) sequencing to examine viral and fungal communities, respectively [22,31,33]. A recent Spain-based clinical trial is further exploring the association of breast cancer with bacterial, viral, fungal, and archaeal dysbiosis in both gut and breast tissues [104,105]. Broadening microbiota signatures in association with breast cancer will add complexity in analyzing data and comparing findings across studies but may potentially allow increased stratification of signatures across different breast cancer sub-types, pathological parameters and biomarker development.

The use of the microbiota as an additional tool for breast cancer diagnosis and association with prognosis factors is an exciting avenue to be explored. The aforementioned studies that use machine learning to elicit a breast “oncobiome” signature that is able to discriminate between healthy and breast cancer patients or tissues demonstrate the potential for using specific gut microbiota signatures for breast cancer diagnosis. These models have shown high accuracy; however, many were validated using internal datasets rather than independent cohorts, which may overestimate their performance and limit real-world applicability. Moreover, differences in microbial markers between pre- and postmenopausal women indicate the need for stratified diagnostic panels.

Most tumor microbiome studies are cross-sectional, limiting our understanding of dynamic changes over time or in response to therapy. Existing studies have primarily focused on chemotherapy response and side effects or skin microbiota in relation to radiodermatitis [42,81,106]. Moreover, understanding how the microbiota influences cancer therapy, including its impact on treatment response and patient survival, could enable earlier interventions, such as dietary modifications, probiotics, or antibiotics, to optimize therapeutic outcomes and reduce treatment-related side effects.

Invasive sampling methods such as tissue biopsies restrict the ability to monitor breast tissue microbiota over time. Therefore, non-invasive sample types, such as feces, oral swabs, vaginal secretions, blood, and NAF, represent promising alternatives. Among these, NAF is particularly valuable for local breast microbiome analysis due to its minimally invasive collection and high patient tolerability [107,108]. Emerging evidence also supports the relevance of circulating microbes in blood, potentially reflecting microbiota translocation via the bloodstream. The presence of oral-associated taxa such as *Porphyromonas* and *Fusobacterium* in breast tissue supports the hypothesis that the circulatory system may facilitate microbial movement between body sites. This raises the possibility of using blood as a real-time indicator of disease-associated microbiota. However, further research is needed to elucidate the mechanisms of microbial translocation across body niches and to determine their potential for use in screening and disease monitoring.

Moving forward, efforts should be made to standardize the reporting between studies in the interest of conducting more robust microbiota research. In support of this, the Strengthening The Organization and Reporting of Microbiome Studies (STORMS) checklist was released in 2021 as a guide for researchers in the preparation and conducting of microbiota studies [109]. In addition, future studies should address the gap in understanding the connection between microbes and function in breast cancer by using multi-omics approaches (metagenomics, meta-transcriptomics, meta-proteomics, and metabolomics) to integrate various data layers. These approaches together provide a more comprehensive view to shed light on the biological activity of the microbiome in the context of breast cancer [98].

## 6. Conclusions

While breast cancer microbiome research is rapidly evolving, significant challenges remain in standardizing methods, validating biomarkers, and translating findings into clinical tools. Future studies should prioritize rigorous experimental design, include ethnically and geographically diverse populations, and incorporate functional and longitudinal data. The use of non-invasive sampling methods for longitudinal microbiota monitoring holds considerable promise. These approaches could support the development of personalized treatment strategies incorporating microbiota modulation to improve therapeutic response and minimize adverse effects. Integration of non-invasive sampling, multi-kingdom microbial profiling, and mechanistic validation will be crucial for advancing microbiota-based strategies in breast cancer prevention, diagnosis, and therapy.

## Authors Contributions

A.Y.W.W., I.P., C.A., A.C. and R.S. devised the main conceptual ideas, A.Y.W.W., G.B. and R.S. wrote and prepared the original draft manuscript with inputs from all authors, all authors were involved reviewing and editing the manuscript, C.A. and R.S. supervised the writing of the manuscript. All authors have read and agreed to the published version of the manuscript.

## Figures and Tables

**Table 1:** Characteristics of studies of the microbiota in relation to breast cancer.

Study (Year)	Methodology for Sequencing/Taxonomic Assignment	Cohort	Niche(s)	Samples	Study Design
Zhu et al. (2018) [10]	Shotgun metagenomic sequencing/IGC by bowtie2	China	Gut (stool)	62 breast cancer patients (18 pre-, 44 postmenopausal), 71 control patients (25 pre-, 46 postmenopausal)	Analysis of gut microbiota in pre- and postmenopausal women
Hou et al. (2021) [11]	16S rRNA sequencing (V3–V4)/OTUs Greengenes 13.8 and BaseSpace RDP	China	Gut (stool)	200 breast cancer patients (100 pre-, 100 postmenopausal), 67 age-matched controls (50 pre-, 17 postmenopausal)	Profiling menopausal-specific gut microbiota in breast cancer
Ma et al. (2020) [12]	16S rRNA (variable region unspecified)/Shanghai Applied Protein Technology	China	Gut (stool)	25 breast cancer, 25 benign breast disease patients	Comparative analysis of gut bacteria and blood metabolites
Zeber-Lubecka et al. (2024) [13]	Shotgun metagenomic sequencing/MetaPhlAn3 v 3.0.13	Poland	Gut (stool)	88 breast cancer patients (47 pre-/peri-, and 41 postmenopausal), 86 controls (51 pre-/peri-, 35 postmenopausal)	Association between breast cancer and gut microbiota
Goedert et al. (2015) [14]	16S rRNA gene sequencing (V3–V4)/OTUs to RDP using the QIIME pipeline	USA	Gut (stool)	48 postmenopausal breast cancer, 48 controls; 85.4% non-Hispanic white in both groups	Case–control study
Byrd et al. (2021) [15]	16S rRNA sequencing (V4)/ASVs to the SILVA database using the DADA2 pipeline 1.2.1	Ghana	Gut (stool)	379 breast cancer patients, 102 benign disease patients, 414 population-based controls	Comparative analysis of fecal microbial profiles
Yang et al. (2021) [16]	16S rRNA gene sequencing (V4)/OTUs to the Greengene database via the QIIME 1.9.1 pipeline	China	Gut (stool)	83 malignant, 19 benign breast tumor patients	Comparative analysis of gut microbiota
He et al. (2021) [17]	16S rRNA sequencing (V3–V4)/unknown pipeline	China	Gut (stool)	54 premenopausal breast cancer patients, 28 healthy controls	Analysis of intestinal microflora changes in comparison to healthy controls
Wu et al. (2020) [18]	16S rRNA sequencing (V3–V4)/OTUs to the RDP using the QIIME pipeline	USA	Gut (stool)	37 breast cancer patients; 73% Hispanic, 75% overweight or obese	Associations of gut microbiomes of breast cancer patients with risk factors and tumor characteristics
Feng et al. (2023) [19]	16S rRNA gene sequencing (V3, V4, V3–V4, and V4–V5)/ASVs using the QIIME 2 pipeline	China	Breast tissue (fresh frozen tissues acquired by fine needle aspiration or core needle biopsy), gut (stool), and oral (saliva)	98 patients with different breast cancer statuses (51 luminal A, 17 luminal B, 18 HER2, and 11 triple-negative), 46 patients with benign breast disease	Comparative study of the microbiota across different sites and breast cancer subtypes
Byrd et al. (2018) [20]	16S rRNA gene sequencing (V3–V4)/OTUs using the QIIME 1.9 pipeline to the RDP classifier and Greengenes 13.8 database	USA	Gut (stool), urine, oral (saline wash)	32 PTEN Hamartoma Tumor Syndrome patients, of which 17 have cancer history, and 15 had no cancer history. 87–100% white cohort	Microbiome analysis in PTEN Hamartoma Tumor Syndrome patients
Xuan et al. (2014) [21]	16S rRNA pyrosequencing (V4)/OTUs using the mothur pipeline Bayesian classifier to Greengenes database	USA	Breast tissue (FFPE)	20 breast cancer patients with paired normal adjacent and tumor tissue; 23 healthy patients undergoing reduction mammoplasty	Comparative analysis of tumor and normal adjacent tissue from the same individual, and healthy breast tissue
Hadzega et al. (2021) [22]	RNA sequencing/Kraken2 and MetaPhlan3	Slovakia and China	Breast tissue (fresh frozen tissues)	18 breast cancer patients, 5 healthy patients undergoing breast cosmesis surgery for Slovakian cohort; Database-downloaded data of 73 triple-negative patients and 18 healthy donor samples for Chinese cohort	Comparative analysis of primary tumor tissues of different breast cancer characteristics
Meng et al. (2018) [23]	16S rRNA gene sequencing (V1–V2)/OTUs using RDP classifier with Greengenes 13.8 reference database within QIIME pipeline	China	Breast tissue (fresh frozen tissues acquired by percutaneous needle biopsy)	22 benign, 72 malignant breast cancer patients	Comparative analysis between benign and malignant breast cancer tissues
Costantini et al. (2018) [24]	16S rRNA gene sequencing (V2, V3, V4, V6+V7, V8, and V9)/OTUs using RDP classifier v. 2.11	Italy	Breast tissue (fresh tissues obtained by core needle biopsy and/or surgical excision biopsy)	12 core needle biopsy, 7 surgical excision biopsy tumors and healthy adjacent tissues from 16 breast patients	Characterization of microbiota in core needle biopsies versus surgical excision biopsies, comparison of breast tumor tissues with healthy adjacent tissues
German et al. (2023) [25]	16S rRNA gene sequencing (V1–V2, V2–V3, V3–V4, V4–V5, V5–V7, V7–V9)/ASVs by alignment to the SILVA 138.1 SSU database via VSEARCH within the QIIME2 2021.4 pipeline	USA	Breast tissue (fresh frozen tissue cores)	403 healthy control women, 76 breast cancer patients that donated one or more tissues from tumor biopsies, normal adjacent tissue, or distant metastatic tissues	Identification of optimal 16S rRNA gene variable region, comparative analysis of breast tissue microbial composition and association of microbial dysbiosis to breast cancer risk factors
Tzeng et al. (2021) [26]	16S rRNA gene sequencing (V3–V4, V7–V9)/ASVs by the DADA2 taxonomy classifier to the SILVA database	USA	Breast tissue (fresh frozen tissues)	221 breast cancer patients, 69 patients without breast cancer, and 18 patients without breast cancer that were categorized as high risk	Correlation study between microbiome and prognostic features
Urbaniak et al. (2016) [27]	16S rRNA gene sequencing (V6)/verified OTUs with Greengenes database	Canada	Breast tissue (fresh frozen tissues)	45 breast cancer patients, 13 benign tumor patients, and 23 disease-free patients	Comparative analysis of breast tissue microbiota
Hoskinson et al. (2022) [28]	16S rRNA gene sequencing (V3–V4)/ASVs to the SILVA reference database using the DADA2 pipeline	USA	Breast tissue (fresh frozen tissues)	50 healthy women, 15 “prediagnostic” women who were healthy at sampling and went on to be diagnosed with breast cancer later, 76 breast cancer patients that donated adjacent normal and/or tumor tissue	Comparative analysis of breast tissue microbiota from healthy, prediagnostic, malignant and adjacent normal breast tissue
Wang et al. (2017) [29]	16S rRNA gene sequencing (V3–V4)/OTUs against Greengenes 13.8 database using UCLUST	USA	Breast tissue (fresh frozen tissues), oral (saline rinse), and urine	57 breast cancer patients (tumor and adjacent normal tissue), and 21 healthy women (two tissue samples, one from each breast)	Comparison of breast tissue, oral, and urinary microbiota with breast cancer and clinical-pathologic features
Esposito et al. (2022) [30]	16S rRNA gene sequencing (V4–V6)/ASVs in BioMaS against the RDP 11.5 database	Italy	Breast tissue (fresh frozen tissues)	Tumoral and adjacent non-tumoral tissue from 34 women with breast cancer	Comparison of microbiota composition of paired tumoral and adjacent non-tumoral tissue
Banerjee et al. (2018) [31]	PathoChip Array	USA	Breast tissue (FFPE)	Breast tissue from different breast cancer subtypes (50 ER+ or PgR+, 34 HER2+, 24 ER+ PgR+ HER2+, and 40 triple-negative), and 20 normal breast tissue controls	Study of microbial (bacterial, viral, fungal, and parasitic) signatures associated with different breast cancer subtypes
Desalegn et al. (2023) [32]	16S rRNA gene sequencing (V4)/ASVs using RDP’s Training Set 16 (11.5) database via the DADA2 pipeline	Ethiopia	Breast tissue (fresh frozen tissue)	50 breast tumors and 50 paired normal adjacent tissues from breast cancer patients	Comparative analysis of breast tissue microbiota between tumor and normal adjacent tissues in Ethiopian women
Banerjee et al. (2021) [33]	PathoChip Array	USA	Breast tissue (FFPE)	95–105 breast tissue samples each for the different breast cancer subtypes (ER+ or PgR+, HER2+, ER+ PgR+ HER2+, and triple-negative), 20 matched control samples, and 68 non-matched control samples	Study of microbial (bacterial, viral, fungal, and parasitic) signatures associated with different breast cancer subtypes, and association to disease outcome
Chan et al. (2016) [34]	16S rRNA gene sequencing (V4)/OTUs using mothur pipeline RDP classifier training set v14	USA	Breast (NAF) and skin control swabs	Nipple aspirate fluid from 25 breast cancer survivors and 23 healthy control women	Characterization of nipple aspirate fluid microbiome
Abstract from Jiwa et al. (2022) [35]	16S rRNA gene sequencing (variable region unspecified)/ASVs (pipeline not specified)	UK	Breast (NAF), with nipple, breast and arm skin as controls	Both breasts of patients were sampled for nipple aspirate fluid, resulting in samples from 23 normal breasts and 22 breasts with tumor	Characterization of nipple aspirate fluid microbiota
Hieken et al. (2016) [36]	16S rRNA gene sequencing (V3–V5)/OTUs to Greengenes 13.5 reference database using the IM-TORNADO pipeline	USA	Breast tissue (fresh frozen tissues)	Aseptically collected normal adjacent breast tissue, skin tissue, and skin swab from patients with benign and malignant breast disease	Comparative study of aseptically collected breast tissue, skin tissue and skin swabs in benign and malignant disease
Thyagarajan et al. (2020) [37]	16S rRNA gene sequencing (V3–V4)/OTUs using the RDP classifier against the Greengenes database	USA	Breast tumor tissue (fresh frozen tissues)	Breast tumor tissue and normal adjacent tissue from a total of 23 white non-Hispanic (17 triple-positive, and 6 triple-negative breast cancer) and 10 black non-Hispanic (7 with triple-positive, 3 with triple-negative breast cancer) that were racial identity-confirmed through ancestry analysis	Comparative analysis of racial differences in breast tumor microbiome, and the differences between triple-positive and triple-negative breast cancer
Balmaganbetova et al. (2021) [38]	Femoflor reagent kit (qPCR)	Kazakhstan	Vagina	278 women with breast cancer (147 luminal A, 57 luminal B, 26 HER2+, 48 triple negative) that comprised 174 patients that received combination therapy during the study and 104 patients that had breast cancer 2–4 years ago	Comparative analysis of vaginal microbiota in women with breast cancer
Peng et al. (2024) [39]	16S rRNA gene sequencing (V3–V4)/ASVs against the SILVA 138 database using the QIIME2 pipeline	China	Blood	107 breast cancer patients and 107 healthy controls	Comparison and correlation of blood microbiota and microbial metabolites between healthy controls and breast cancer patients
An et al. (2023) [40]	16S rRNA gene sequencing (V3–V4)/OTUs using UCLUST against the SILVA 132 database via QIIME 1.9.1 pipeline	South Korea	Blood (isolated bacterial extracellular vesicles)	96 patients with breast cancer and 192 healthy controls	Blood microbiota data for the development of a breast cancer diagnostic algorithm using blood microbiota patterns
Shi et al. (2019) [41]	16S rRNA gene sequencing (V3–V4)/OTUs using RDP classifier v2.2 via the UPARSE pipeline	China	Gut (stool)	80 breast cancer patients	Analysis of gut microbiota and its diversity in breast cancer in correlation to tumor infiltrating lymphocyte status
Klymiuk et al. (2022) [42]	16S rRNA gene sequencing (V4–V5)/ASVs against the SILVA 138 database via the QIIME2 pipeline	Austria	Oral (saliva)	Breast cancer patients with non-metastatic breast cancer undergoing chemotherapy, samples were obtained over three timepoints	Analysis of chemotherapy-associated changes in oral microbiome

Abbreviations used: ASV, amplicon sequence variant; BioMaS, Bioinformatic analysis of Metagenomic AmpliconS; DADA2, Divisive Amplicon Denoising Algorithm 2; ER, estrogen receptor; FFPE, formalin-fixed paraffin-embedded tissues; HER2, human epidermal growth factor receptor 2; IGC, integrated reference catalog of the human gut microbiome; IM-TORNADO, Illinois Mayo Taxon Organization from RNA Dataset Operations; MetaPhlAn, Metagenomic Phylogenetic Analysis; NAF, nipple aspirate fluid; OTU, operational taxonomic unit; PTEN, Phosphatase and TENsin homolog deleted on chromosome 10; QIIME, Quantitative Insights Into Microbial Ecology; PgR, progesterone receptor; qPCR, quantitative polymerase chain reaction; RDP, Ribosomal Database Project; VSEARCH, vectorized search.

**Table 2:** Diagnostic performance of microbiota signatures used to differentiate women with and without breast cancer.

Study (Year)	Niche	Breast Cancer Microbiota Signature Utilized	AUC
Zhu et al. (2018) [10]	Gut (stool)	*Fusobacterium varium*, *Shigella_sp_D9*, *Desulfovibrio piger*, *Escherichia_sp_1_1_43*, *Shigella sonnei*, *Eubacterium eligens*, *Escherichia_sp_3_2_53FAA*, *Vibrio cholerae*, *Acinetobacter baumannii*, *Proteus mirabilis*, *Fusobacterium nucleatum*, *Campylobacter concisus*, *Escherichia coli*, and *Porphyromonas uenonis*	87.25% (95% CI 77.57–93.47%) on the training sample cohort of postmenopausal patients, 72% (95% CI 56.01–88.44%) on the test sample cohort consisting of both pre- and postmenopausal patients
Hou et al. (2021) [11]	Gut (stool)	Premenopausal: *Bacteroides fragilis*, *Anaerostipes*, *Haemophilus parainfluenzae*, *Sutterella*, *Faecalibacterium prausnitzii*, *Bifidobacterium adolescentis*, *Bifidobacterium longum*, *Bifidobacterium bifidum*, *Ruminococcus gnavus*, *Rothia mucilaginosa* Postmenopausal: *Klebsiella pneumoniae*, *Haemophilus parainfluenzae*, *Sutterella*, *Akkermansia muciniphila*, *Phascolarctobacterium*, *Ruminococcus gnavus*, *Rothia mucilaginosa* Both pre- and postmenopausal: *Haemophilus parainfluenzae*, *Sutterella*, *Faecalibacterium prausnitzii*, *Ruminococcus gnavus*, *Rothia mucilaginosa*	0.826 for premenopausal women, 0.887 for postmenopausal women, 0.791 for both pre- and postmenopausal women
Zeber-Lubecka et al. (2024) [13]	Gut (stool)	Premenopausal: *Bacteroides vulgatus*, *Eubacterium eligens*, *Bifidobacterium adolescentis*, *Parabacteroides distasonis*, *Instestinimonas butyriciproducens*, *Alistipes finegoldii*, *Gordonibacter pamelaeae*, *Ruthenibacterium lactatiformans*, *Gemmiger formicilis*, *Alistipes shahii*, *Roseburia intestinalis*, *Collinsella intestinalis*, *Pseudoflavonifractor* sp. *An 194*, *Enterorhabdus caecimuris*, *Faecalibacterium prausnitzii* Postmenopausal: *Alistipes finegoldii*, *Faecalibacterium prausnitzii*, *Barnesiella intestinihominis*, *Parabacteroides distasonis*, *Dorea longicatena*, *Alistipes putredinis*, *Eubacterium ramulus*, *Alistipes indistinctus*, *Coprobacter fastidious*, *Eubacterium ventriosum*, *Eubacterium* sp. *CAG 38*, *Agathobaculum butyriciproducens*, *Ruminococcus bromii*, *Enterorhabdus caecimuris*, *Asacharobacter celatus*	0.866 (95% CI 0.717–1.000) in premenopausal women, 0.810 (95% CI 0.579–1.000) in postmenopausal women
Esposito et al. (2022) [30]	Breast tissue	*Propinionibacterium acnes*, *Acinetobacter johnsonii*, *Bacillus* sp. *YDWLR1*, *Pseudomonas putida*, *Actinetobacter junii*, *Xanthomonas citri*, *Diaphorobacter*, *Staphylococcus aureus*, *Staphylococcus epidermidis*, *Pseudomonas stutzeri*, and *Enterobacter aerogenes*	89% in their patient cohort (reported as diagnosis accuracy)
An et al. (2023) [40]	Blood	*Enterobacter*, *Bacteroides*, *Kluyvera*, *Pseudomonas*, *Parabacteroides*, *Enterobacter*, *Pseudomonas*, *Bacteroides*, *Staphylococcus*, *Acinetobacter*, and *Corynebacterium 1*	0.978–0.996 in their cohort of training and test set at an 80:20 ratio

Abbreviations used: AUC, Area Under the Receiver Operating Characteristic Curve; 95% CI, 95% confidence interval.
